# Understanding the Dual Challenge: Adverse Drug Reactions and Adherence in Rheumatoid Arthritis Treatment

**DOI:** 10.7759/cureus.76712

**Published:** 2025-01-01

**Authors:** Aishwarya Nikose, Swati Patil, Neha Kadhe

**Affiliations:** 1 Pharmacology and Therapeutics, Lokmanya Tilak Municipal Medical College and General Hospital, Sion, Mumbai, IND; 2 Clinical Pharmacology, Seth Gordhandas Sunderdas Medical College, Mumbai, IND; 3 Pharmacology and Therapeutics, Lokmanya Tilak Municipal Medical College and Hospital, Sion, Mumbai, IND

**Keywords:** adr- adverse drug reactions, cqr5, folic acid supplementation, medication adherence, methotrexate intolerance, miss methotrexate intolerance severity score, rheumatoid arthriitis

## Abstract

Objectives: The primary aim of this study was to investigate the real-world occurrence of various adverse drug reactions (ADRs) and assess medication adherence among patients using disease-modifying antirheumatic drugs (DMARDs). Additionally, the study sought to evaluate the degree of methotrexate intolerance among these patients.

Methods: This cross-sectional, observational study was conducted at a tertiary care hospital and involved 100 adult patients diagnosed with rheumatoid arthritis (RA) who were currently undergoing treatment with methotrexate. The study systematically recorded all adverse drug reactions reported by the patients as well as those identified through laboratory tests. Medication adherence was measured using the Compliance Questionnaire Rheumatology (CQR5) and both intentional and non-intentional non-adherence questionnaires. The methotrexate intolerance was assessed using the Methotrexate Intolerance Severity Score (MISS).

Results: Out of the total 74 reported ADRs, gastrointestinal were the most common, accounting for 62 (76.53%) of cases, followed by hematological problems at 13 (16.07%), with other types of ADRs comprising the remaining. Despite the prevalence of gastrointestinal ADRs, the study found high levels of medication adherence among the patients. Additionally, the methotrexate intolerance was relatively low, which may be attributed to the use of folic acid supplementation by all participants.

Conclusion: Gastrointestinal adverse drug reactions were the most frequently observed side effects in this study. Despite these reactions, patients demonstrated a high level of adherence to methotrexate therapy. The factors contributing to this adherence- such as patient counseling, disease knowledge, or the duration of the disease- merit further investigation to understand their impact on treatment outcomes.

## Introduction

Rheumatoid arthritis (RA) is a chronic inflammatory disease that affects patients' social, emotional, and physical well-being. The prevalence of rheumatoid arthritis in India is 0.75% with women approximately 2.5 times more likely to develop rheumatoid arthritis than men [1].

Disease modifying anti-rheumatic drugs (DMARDs), both conventional synthetic (csDMARDs) and biologicals (bDMARDs), have proven to be effective in reducing or reversing symptoms, preventing disability, and preventing progression of joint damage. DMARDs can be used as monotherapy or as combination therapy. Methotrexate, sulfasalazine, azathioprine, cyclosporine, and leflunomide are commonly prescribed non-biological DMARDs. Tumor necrosis factor (TNF) inhibitors (etanercept, adalimumab, infliximab, golimumab, certolizumab pegol), T cell costimulatory inhibitors (abatacept), IL-6 receptor inhibitors (tocilizumab, sarilumab), and anti-CD20 antibody (rituximab) are biological DMARDs used for rheumatoid arthritis.

The most common documented adverse drug reactions (ADRs) to DMARDs are gastrointestinal in nature ranging from mild abdominal discomfort to severe vomiting, diarrhoea, oral ulcer and nausea. Other common types of adverse drug reactions include chest tightness, anaemia, fatigue, and alopecia, hepatotoxicity, and renal problems.

Methotrexate is indicated as first-line therapy for rheumatoid arthritis and occupies a prominent position in many guidelines and recommendations for the treatment of rheumatic diseases. In spite of its effectiveness and low cost, methotrexate can cause gastrointestinal side effects, such as abdominal pain, nausea, vomiting and diarrhoea [2]. Although folic acid supplementation during methotrexate treatment may reduce such effects, many patients discontinue treatment, something that negatively impacts disease control and quality of life. The Methotrexate Intolerance Severity Score (MISS) has been used to assess methotrexate-induced gastrointestinal adverse effects.

Adherence to long-term therapy can be defined by the extent to which a person’s behaviour, i.e., taking medication, following a diet and/or executing lifestyle change, corresponds with agreed recommendations from a healthcare provider. Adherence rate to DMARDs in patients with rheumatoid arthritis is very low and has varied from 16.4% to 76.9% [3]. Non-adherence results in higher disease activity, radiographic damage, disability, lower quality of life, and higher healthcare cost. Multiple factors have been shown to affect medication adherence in patients with rheumatoid arthritis. However, there are varied results about the influences of patient and disease characteristics on adherence [4].

Hence this study was planned to assess the prevalence and spectrum of adverse drug reactions to DMARDs in patients with rheumatoid arthritis with a special focus on methotrexate intolerance and non-adherence to treatment.

## Materials and methods

Study design and approval

A single-centre, cross-sectional, observational study was conducted in the Rheumatology outpatient department (OPD) of a tertiary care hospital in India for a period of 18 months, starting from May 2021 to November 2022. Ethical approval was obtained from the Institutional Ethics Committee (IEC) before start of the study (IEC No: IEC/432/21). A Written Informed Consent was obtained from the study participants prior to enrolment.

Sample size calculations

A base sample size of 72 was determined assuming a 0.75% prevalence of rheumatoid arthritis with a Z score of 1.96 is used with sampling error [1]. Based on literature published study and feasibility, 100 patients were enrolled in the study.

Inclusion and exclusion

Adult patients (aged more than 18 years), with a diagnosis of RA by a physician as per American College of Rheumatology criteria 2020, either on methotrexate monotherapy or combination with other DMARDs for at least three months, willing to give consent and be a part of the study were included. Patients having any history of psychiatric illness were excluded.

Methodology

All patients satisfying the inclusion and exclusion criteria were enrolled after seeking informed consent. The details were obtained from the information derived from the patient by semistructured interview and medical records and documented in the case record form (CRF) by the principal investigator.

Outcome parameters

The evaluated outcome parameters were ADRs reported by patients and from laboratory records in the last one year from medical records, adherence score using Compliance Questionnaire for Rheumatology 5 (CQR5) and unintentional and intentional adherence questionnaire and methotrexate intolerance using MISS by a semistructured interview with the patient [5-8].

Statistical analysis

All the data were collected, entered and analysed in Microsoft Excel version 2016 (Redmond, WA, USA). Continuous variables were expressed as mean ± standard deviation (SD), whereas categorical variables were expressed as percentages and frequencies.

## Results

Demographic details

Demographic details were presented with respect to presence or absence of ADR to DMARDS in Table 1. 

**Table 1:** Demographic details of participants n= Number of participants ADR: adverse drug reaction

Parameters	Total patients	Patients with ADR (n=74)	Patients without ADR (n=26)
	(n=100)		
Age in years:			
a. Median	47	49	43
b. IQR	40 - 54	41 – 54.75	37.25 - 49.5
c. Minimum age	22	28	22
d. Maximum age	71	71	71
Gender:			
a. Males	10 (10%)	7 (9.45%)	3 (11.53%)
b. Females	90 (90%)	67 (90.55%)	23 (88.47%)
Socioeconomic status:			
a. Lower	9 (9%)	6 (8.11%)	3 (11.53%)
b. Upper lower	63 (63%)	47 (63.52%)	16 (61.54%)
c. Lower middle	24 (24%)	18 (24.32%)	6 (23.08%)
d. Upper middle	4 (4%)	3 (4.05%)	1 (3.85%)
Disease severity:			
a. Moderate	26 (26%)	20 (27.02%)	6 (23.08%)
b. High	74 (74%)	54 (72.98%)	20 (76.92%)
Disease duration in months:			
a. Median	60	60	48
b. IQR	24 – 96	36-96	16.5 – 81
c. Minimum Duration	4	4	4
d. Maximum Duration	384	384	240
History of smoking:			
a. Yes	5 (5%)	4 (5.40%)	1 (3.84%)
b. No	95 (95%)	70 (94.60%)	25 (96.16%)
Comorbidities:			
a. Yes	59 (59%)	47 (63.51%)	12 (46.15%)
b. No	41 (41%)	27 (36.49%)	14 (53.85%)
History of peptic ulcer disease:			
a. Yes	72 (72%)	67 (90.54%)	5 (19.24%)
b. No	28 (28%)	7 (9.46%)	21 (80.76%)

A total of 100 patients were enrolled from the outpatient rheumatology department during the 18-month study period who were on methotrexate. There were 90 females (90%) and 10 males (10%) with a median age of 47 (IQR= 40-54) years. Sixty-three percent of patients were from the upper lower class as per the modified Kuppuswamy socioeconomic status scale 2020.

Severity of RA was assessed at the time of enrolment of patients in the study by interviewing patients using Rheumatoid Arthritis Disease Activity Index-5 (RADAI-5) and 74 patients had severe disease. Fifty-three percent of patients reported a disease duration of 0-60 months (0-5 years), whereas 33% reported a disease duration of 61-120 months (6-10 years), with median disease duration of 60 months (5 years) (IQR = 24-90 months).

History of comorbid conditions

Among enrolled patients, 59 patients reported comorbid conditions, and 19 patients reported more than one comorbidity. Comorbidities seen in patients were hypertension, hypothyroidism, diabetes mellitus, interstitial lung disease, dyslipidaemias, etc. Out of 100, 72 patients gave a history of symptoms suggestive of peptic ulcer disease and 64 (88.89%) patients were started on proton pump inhibitors like pantoprazole for treatment and prevention of further progression of peptic ulcer disease.

Drug and ADR details

For the management of rheumatoid arthritis, DMARDs, corticosteroids, non-steroidal anti-inflammatory drugs (NSAIDs) and folic acid derivatives were prescribed (Table 2). 

**Table 2:** Details of drugs used in management of rheumatoid arthritis in this study. DMARD: Disease modifying anti-rheumatic drugs NSAIDs: Non-steroidal anti-inflammatory drugs ATC Code: Anatomical Therapeutic Chemical Code PO: Per Oral IM: Intramuscular SC: Subcutaneous MTX: Methotrexate

Drug class	Drug	ATC Code	Dose (mg)	Mean dose (Mean ± SD)	Route	Number of Participants (n)
DMARD	MTX	L04AX03	5	(7.4 ± 2.245)	PO	100
	MTX	L04AX03	7.5		PO	
	MTX	L04AX03	10		PO	
	MTX	L04AX03	15		IM	
	MTX	L04AX03	20		SC	
DMARD	Hydroxychloroquine	P01BA02	200	(342.55 ± 90.97)	PO	94
	Hydroxychloroquine	P01BA02	400		PO	
DMARD	Sulfasalazine	A07EC01	500	(505.494 ± 52.414)	PO	91
	Sulfasalazine	A07EC01	1000		PO	
DMARD	Leflunomide	L04AA25	10	(14.375 ± 6.232)	PO	9
	Leflunomide	L04AA25	20		PO	
	Leflunomide	L04AA25	25		PO	
DMARD	Azathioprine	L04AX01	50		PO	2
Corticosteroids	Prednisolone	S02BA03	2.5	(6.382 ± 4.237)	PO	16
	Prednisolone	S02BA03	5		PO	
	Prednisolone	S02BA03	10		PO	
	Prednisolone	S02BA03	20		PO	
Corticosteroids	Deflazacort	H02AB13	6		PO	1
NSAIDs	Indomethacin	M02AA23	25		PO	86
Folic acid derivatives	Folic acid	B03BB01	5		PO	100

Methotrexate was the most prescribed DMARD at twice a week with a mean dose of 7.4 ± 2.245 mg as monotherapy or as a combination therapy. The routes of administration for methotrexate were subcutaneous (SC) in two patients, intramuscular (IM) in one patient and the rest via oral route with a median therapeutic duration of 60 months. Folic acid was prescribed in all patients for five days a week.

Of the 100 patients, 74 reported adverse drug reactions to DMARDs, with 69 patients (93.25% of those reporting ADRs) experiencing at least one adverse event. The most frequently reported adverse drug reactions were gastrointestinal (62, 76.53%), which included gastritis (59, 72.83%) and oral ulceration (3, 3.70%) followed by haematological adverse drug reactions (13, 16.07%) (Table 3). Thirteen (17.56%) patients discontinued the drug due to adverse reactions, three were substituted with other DMARDs and dose reduction was done in two patients and three were hospitalized for ADR management.

**Table 3:** Details of patient reported adverse drug reactions and adverse drug reactions from laboratory records observed in the last one year in patients (n=81). n: Number of Adverse drug reactions WHO-ART: World Health Organization Adverse Reaction Terminology MedDRA: Medical Dictionary for Regulatory activities ADRs: Adverse Drug Reactions

WHO ART Term/ MedDRA Preferred term	System Organ Class (Soc)	Number of adverse drug reactions (n)	Percentage (%)	
Gastritis	Gastrointestinal disorders	59	72.83	
Leucopenia	Blood and lymphatic system disorders	8	9.91	
Hepatitis	Hepatobiliary disorders	5	6.17	
Thrombocytopenia	Blood and lymphatic system disorders	3	3.70	
Ulcerative stomatitis/Oral ulceration	Gastrointestinal disorders	3	3.70	
Anaemia	Blood and lymphatic system disorders	2	2.46	
Dry Cough/Cough	Respiratory, thoracic and mediastinal disorders	1	1.23	
Total		81		100

Adherence to DMARDs

Adherence to DMARDs was assessed by semi-structured interviews using CQR5 and intentional and unintentional non-adherence questionnaires. Among 100 patients, 10 patients were non-adherent intentionally and 11 patients unintentionally. Methotrexate intolerance in patients who are on methotrexate therapy was assessed by a semi-structured interview using MISS during the last 15 days of enrolment and 92% of patients were not intolerant to methotrexate.

## Discussion

Rheumatoid arthritis is a complicated and dynamic condition with equally complex therapeutic choices. The severity of the condition, the site of injury, comorbidities or contraindications, the cost of the therapy, and whether monotherapy or drug combinations are necessary factors for deciding treatment regimen.

As per American College of Rheumatology guidelines for the treatment of rheumatoid arthritis 2021, the main goal of management of rheumatoid arthritis is to control disease activity and slow the rate of joint damage, in addition to reducing pain, inflammation, stiffness and complications. The drugs used for management of rheumatoid arthritis are broadly divided into two types: drugs that provide disease-modifying therapy and drugs for symptomatic treatment. Toxicity may be a limiting factor with the wide range of therapeutic choices available with DMARDs [9].

Methotrexate monotherapy or in combination with other DMARDs is still regarded as an important treatment for rheumatoid arthritis and the most bearable in long-term therapy, despite the development of novel biological agents. Methotrexate along with other DMARDs can cause a variety of adverse drug reactions which includes gastrointestinal, haematological, hepatological, cutaneous, etc. So, there is a need to study the spectrum of adverse drug reactions to DMARDs. Despite being an important medicine in the treatment of rheumatoid arthritis, methotrexate can cause gastrointestinal intolerance [10]. Gastrointestinal adverse drug reactions are a primary cause of therapeutic non-adherence. Therefore, study into methotrexate intolerance and therapy adherence is necessary.

In this study, we evaluated the real-world occurrence and spectrum of adverse drug reactions to DMARDs in patients with rheumatoid arthritis. We also evaluated adherence to DMARDs using CQR-5 and intentional and unintentional non-adherence questionnaires [5,7]. We also evaluated methotrexate intolerance using MISS questionnaire in rheumatoid arthritis patients [11,12].

Demographic details

The median age of the 100 rheumatoid arthritis patients evaluated in this study was 47 years (interquartile range: 40-54), which is equivalent to the mean age of rheumatoid arthritis in India, which is 48 years [13]. The incidence of rheumatoid arthritis was more in female patients (90%) than male patients (10%) in this study. As per population surveys developed by the World Health Organization - International League of Associations for Rheumatology (WHO-ILAR) Community Oriented Program for Control of Rheumatic Diseases (COPCORD), females are more prone to develop rheumatoid arthritis than males [14-16].

Use of NSAIDs and prednisolone are exacerbating factors for peptic ulcer disease and that is why incidence of peptic ulcer disease was more in the observed population in the current study [17-21]. Out of 72 patients, 64 (88.89%) patients were started on proton pump inhibitors like pantoprazole for treatment and prevention of further progression of peptic ulcer disease.

Drug details

For the management of rheumatoid arthritis, DMARDs are first-line therapy as per American College of Rheumatology (ACR) guidelines, along with NSAIDs, glucocorticoids and folic acid derivatives [22].

In the current study, DMARDs like methotrexate (100), hydroxychloroquine (94), sulfasalazine (91), leflunomide (9) and azathioprine (2) were given. Methotrexate (100) was prescribed at a dose of 5 mg (19), 7.5 mg (76), 10 mg (2), 15 mg (1) and 20 mg (2) with a frequency of twice a week. In clinical practice, the maintenance dose of methotrexate ranges from 7.5 to 30 mg per week, indicating that patients required individualised dose for optimal disease control. This variation in methotrexate dosage used in our study may be due to the fact that patients included were of varying disease severity and treatment duration [23]. As a folate pathway antagonist, methotrexate acts as an inhibitor of de novo purine and pyrimidine synthesis required for DNA and RNA synthesis, although a number of more subtle mechanisms of action have been proposed when used as a DMARDs. This leads to spectrum of adverse drug reactions to methotrexate even when used in small doses.

All patients were prescribed folic acid, which reduces the adverse effects of methotrexate, a folate antagonist. Folic acid was prescribed five days a week when methotrexate was not administered. Folic or folinic acid greatly lowers the frequency of gastrointestinal side effects and hepatic impairment, resulting in reduction in risk of non-adherence to therapy. Concurrent use of folic acid and methotrexate reduces the clinical efficacy of methotrexate, while folic acid administration, the day after methotrexate, prevents this interaction by inhibiting the competition between folate and methotrexate for absorption [23].

The most commonly prescribed combination therapy along with NSAIDs and glucocorticoids included (triple drug therapy + NSAIDs) i.e., (methotrexate, hydroxychloroquine, sulfasalazine and indomethacin) in 59 patients and it is comparable with available literature [24].

Along with the drugs prescribed for treatment of rheumatoid arthritis, concomitant drugs were also prescribed for prevention of adverse effects of drugs prescribed for treatment of rheumatoid arthritis. Concomitant drugs included pantoprazole, a proton pump inhibitor, and ondansetron, a serotonin (5HT3) antagonist. Pantoprazole (87) was prescribed for prevention of peptic ulcer disease. Ondansetron (5) was prescribed in patients complaining of severe nausea and vomiting after taking drugs prescribed for treatment of rheumatoid arthritis.

Patient-reported adverse drug reactions and adverse drug reactions from laboratory records observed in the last one year in patients

Medications used for treating rheumatoid arthritis that are DMARDs have a history of serious adverse drug reactions. In this study, the most prevalent conditions were gastrointestinal (76.53%), which included gastritis (72.83%) and oral ulceration (3.70%) [25-27]. A study conducted by Salliot et al. states that gastrointestinal adverse events were the most common side effect (52-65%) and had similar incidence whatever the duration of methotrexate [28].

The second most frequent adverse drug reactions were haematological (16.07%) and included leucopenia (9.91%), thrombocytopenia (3.70%), and anaemia (2.46%), which was consistent with the literature at the time [26]. Additionally, hepatitis (6.17%) and cough (1.23%) were noted.

Combination medication increased the risk of adverse drug reactions higher than monotherapy, and this finding is consistent with another study [29]. Folic acid, which is known to lessen methotrexate toxicity, was administered to all study participants. The incidence of methotrexate causing gastritis, leucopenia, neutropenia, anaemia, and oral ulceration is common and rare, respectively, according to SmPC guidelines. All adverse drug reactions were treatable and reversible. No adverse drug reactions were fatal, and no medication errors were encountered during the study.

Adherence to disease-modifying anti-rheumatic drugs

Medication adherence is crucial for intended clinical outcomes because therapeutic success depends on both drug efficacy and adherence. Data was collected from patients by having semi-structured interviews using CQR-5 and data analysed by using CQR5 adherence calculator. In the current study, 88% of patients showed high adherence to DMARDs and 12% showed low adherence by using CQR-5. Improvements in adherence are also influenced by views about medications and how people perceive their illnesses, but in this study, we did not evaluate these factors, thus there was insufficient data to measure adherence.

Intentional nonadherence suggests decision-driven behaviour, based on patient perceptions of illness and beliefs in prescribed therapies. Unintentional nonadherence suggests a questionable capacity for taking medication, including forgetfulness, poor manual dexterity, medication loss, or non-affordability. Both sets of adherence measures (unintentional and intentional) were adapted from validated methods published in the peer-reviewed literature. Subjects responded to three questions pertaining to intentional and unintentional nonadherence each during the past two weeks and 10% were non-adherent intentionally and 11% un-intentionally.

A 10-year longitudinal study with rheumatoid arthritis patients in Denmark by Thurah et al. revealed an adherence rate of 80% which is comparable to current study. Folic acid supplementation, which is now considered standard of care, can boost adherence to methotrexate and in this study, all patients took folic acid supplements, resulting in high adherence [8].

Intolerance to methotrexate

Gastrointestinal adverse drug reactions contributed 76.53% of total reported adverse drug reactions. There is a tendency to develop intolerance to medication because of gastrointestinal adverse drug reactions. Methotrexate intolerance was assessed by using the MISS questionnaire [8].

According to the MISS questionnaire used in the current investigation, 8% of the patients were intolerant to methotrexate. Out of 100 recruited patients, 48 reported experiencing nausea, 12 reported vomiting, 60 reported abdominal discomfort, and one reported behavioural symptoms. The MISS tool was unable to identify cases in this study since pantoprazole was being taken by 87 individuals. All patients in this study took folic acid which is known to reduce gastrointestinal intolerance to methotrexate.

The MISS is validated to assess methotrexate intolerance in juvenile idiopathic arthritis patients and that’s why it provides a structured framework for assessment of methotrexate intolerance in adults. However, compared to children, behavioural symptoms in adults may not be as noticeable. The MISS should be modified and validated for adults with rheumatoid arthritis. This may be beneficial for prevention of discontinuation of effective therapy for rheumatoid arthritis.

Limitations of the study

The inclusion of patients from one institution raises the likelihood of selection bias. Although this study's sample size was modest compared to clinical trials and it was carried out in a single location, it has the advantage of providing information about actual instances of adverse drug reactions in the real world. As it is an observational, cross-sectional study, it cannot determine how well adherence is improving and the causal relationship between the disease and outcomes, which call for long-term follow-ups. Association of ADR with age, number of DMARDs, and comorbidities need to be evaluated. As all parameters required for assessment of adherence to medication are not studied, the incidence of adherence to DMARDs is high in this study. This study does not identify risk factors for methotrexate intolerance, nor does it show how frequently patients stop using methotrexate or switch to another medicine as a result of methotrexate intolerance.

## Conclusions

Rheumatoid arthritis is a chronic inflammatory disorder, with DMARDs serving as the primary treatment, among which methotrexate is the most commonly used. Gastritis was the most common adverse drug reaction. Despite use of folic acid in all patients, gastrointestinal ADRs were seen commonly. Patients showed good adherence to therapy, whether counselling, good knowledge of disease or longer duration of disease responsible, needs further evaluation. Patients receiving pantoprazole therapy limits the use of MISS for assessing intolerance to methotrexate. In our setting we observed low dose methotrexate in rheumatoid arthritis patients, spectrum of ADR in other setting requiring higher doses of methotrexate or routes other than oral need further evaluation.
